# Engineering of Acetate Recycling and Citrate Synthase to Improve Aerobic Succinate Production in *Corynebacterium glutamicum*

**DOI:** 10.1371/journal.pone.0060659

**Published:** 2013-04-08

**Authors:** Nianqing Zhu, Huihua Xia, Zhiwen Wang, Xueming Zhao, Tao Chen

**Affiliations:** 1 Key Laboratory of Systems Bioengineering, Ministry of Education, Tianjin University, Tianjin, People’s Republic of China; 2 Department of Biochemical Engineering, School of Chemical Engineering and Technology, Tianjin University, Tianjin, People’s Republic of China; University Paris South, France

## Abstract

*Corynebacterium glutamicum* lacking the succinate dehydrogenase complex can produce succinate aerobically with acetate representing the major byproduct. Efforts to increase succinate production involved deletion of acetate formation pathways and overexpression of anaplerotic pathways, but acetate formation could not be completely eliminated. To address this issue, we constructed a pathway for recycling wasted carbon in succinate-producing *C. glutamicum*. The acetyl-CoA synthetase from *Bacillus subtilis* was heterologously introduced into *C. glutamicum* for the first time. The engineered strain ZX1 (pEacsA) did not secrete acetate and produced succinate with a yield of 0.50 mol (mol glucose)^−1^. Moreover, in order to drive more carbon towards succinate biosynthesis, the native citrate synthase encoded by *gltA* was overexpressed, leading to strain ZX1 (pEacsAgltA), which showed a 22% increase in succinate yield and a 62% decrease in pyruvate yield compared to strain ZX1 (pEacsA). In fed-batch cultivations, strain ZX1 (pEacsAgltA) produced 241 mM succinate with an average volumetric productivity of 3.55 mM h^−1^ and an average yield of 0.63 mol (mol glucose) ^−1^, making it a promising platform for the aerobic production of succinate at large scale.

## Introduction

Succinic acid is considered as one of the most important platform chemicals because of its extensive applications in many industrial fields. It can be converted to a wide range of products including specialty chemicals, food ingredients, green solvents, pharmaceuticals and biopolymers [1], [2]. Currently, succinic acid production is accomplished by chemical synthesis using petroleum-derived maleic anhydride. However, this process requires high temperature, high pressure and heavy metal catalysts, which makes the conversion costly and ecologically questionable [1], [3]. Thus, microbial production of succinate from cheap and renewable carbon sources attracted great interest as more sustainable replacement for conventional petrochemical routes in recent years. In 2004, succinic acid was identified by the U.S. Department of Energy as one of the top 12 building block chemicals that could be produced from renewable resources [4].

Succinate can be produced anaerobically by naturally isolated bacteria and recombinant organisms, such as rumen bacteria *Actinobacillus succinogenes* [5], *Anaerobiospirillum succiniciproducens* [6] and *Mannheimia succiniciproducens* [7], as well as recombinant *Escherichia coli* [8]. The rumen bacteria have shown great potential for industrial succinate production, but some of them are potentially pathogenic or strictly anaerobic and difficult to culture [1]. *Corynebacterium glutamicum*, a rapidly growing Gram-positive soil bacterium that can utilize a broad spectrum of carbon sources, represents another promising platform for succinate production. It does not grow under anaerobic conditions, but has the ability to utilize sugars and excrete lactate, succinate and small amounts of acetate in the presence of additional bicarbonate [9]. Based on this capability, metabolically engineered *C. glutamicum* strains (ATCC 13032 and R) were developed and the culture conditions were optimized for anaerobic production of succinate [9]–[11].

Even though anaerobic processes for succinate production have resulted in high yields and titers, some problems are still to be solved, e.g. the optimal redox balance was limited [12], [13]. The aerobic cell culture conditions were reported to have advantages over anaerobic conditions due to higher biomass generation, faster carbon throughput and product formation [13], [14]. Thus, aerobic platforms were designed and constructed. Several aerobic succinate-producing *E. coli* strains were designed and could achieve high succinate yields using various sugars [12], [14], [15]. The yeasts *Saccharomyces cerevisiae* and *Yarrowia lipolytica* which showed high tolerance toward acidity were also genetically modified for succinate production, but with low volumetric productivities [3], [16]. Recently, Litsanov et al [13] reported that *C. glutamicum* ATCC 13032 lacking the succinate dehydrogenase complex showed greater potential for aerobic succinate production than other producers with equivalent genetic backgrounds. Disruption of all known acetate formation pathways as well as overexpression of anaplerotic pathways resulted in a strain that produced 90 mM succinate with a yield of 0.45 mol (mol glucose) ^−1^ under optimized production conditions [13]. But this strain produced succinate with a low volumetric productivity of 0.77 mM h^−1^ and accumulated acetate with a yield of 0.08 mol (mol glucose) ^−1^ [13]. The acetate accumulation resulted in a waste of carbon source, therefore negatively affecting succinate production.

The aims of this study are to eliminate acetate accumulation as well as to further increase succinate production by *C. glutamicum* ATCC 13032 under aerobic conditions. First, the acetate utilization pathway from *Bacillus subtilis* was introduced into *C. glutamicum* ATCC 13032. Moreover, the native citrate synthase was overexpressed to redirect more carbon flux towards the tricarboxylic acid (TCA) cycle. Also, the effect of overexpression of the succinate exporter *sucE* gene, normally induced under anaerobic conditions, on succinate production was examined. Finally, a succinate titer of 241 mM with a volumetric productivity of 3.55 mM h^−1^ and a yield of 0.63 mol (mol glucose) ^−1^ were achieved by fed-batch cultivations.

## Materials and Methods

### Strains, Plasmids and Media

All strains and plasmids used in this study, their sources and relevant characteristics were given in Table 1. During plasmids construction, *E. coli* DH5α was used and cultured in lysogeny broth complex medium (LB). Plasmid DNA transferring into *C. glutamicum* ATCC 13032 was carried out by electroporation, and the recombinant strains were selected on brain heart infusion-sorbitol (BHIS) agar plates containing 25 µg mL^−1^ kanamycin.

**Table 1:** Bacterial strains and plasmids used in this study.

Strain or plasmid	Relevant characteristics	Reference
Strains		
*C. glutamicum* ATCC13032	Wild-type, biotin auxotrophic	ATCC^a^
Δ*sdhCAB*	Wild-type with deletion of *sdhCAB*	This study
SAZ3	Wild-type with deletions of *ldhA*, *pqo*, *cat* and *pta* and replacement of the native promoters of *pyc* and *ppc*by the *sod* promoter	Unpublished work
ZX1	Strain SAZ3 with deletion of *sdhCAB*	This study
Plasmids		
pK18*mobsacB*	Kan^R^; vector for in frame deletion (RP4 *mob*; *sacB_B.sub._*; *lacZ*α; *OriV_E.c._*)	[33]
pD*sacB*	derived from pK18*mobsacB,* for increasing expression of *sacB* under *P_trc_*	This study
pD*sacB-sdhCAB*	pD*sacB* carrying upstream and downstream regions of *sdhCAB* operon	This study
pEC-xk99E	Kan^R^; *C. glutamicum/E. coli* shuttle vector (*P_trc_*, *lacI* ^q^; pGA1, *OriV_C.g._*, *OriV_E.c._*)	[34]
pEacsA	derived from pEC-XK99E, for overexpression of *acsA* under the control of *P_trc_*	This study
pEacsAgltA	derived from pEC-XK99E, for overexpression of *acsA* and *gltA* under the control of *P_trc_*	This study
pEacsAgltAsucE	derived from pEC-XK99E, for overexpression of *acsA* and *gltA* under the control of *P_trc_*, overexpression of *sucE*under the control of its native promoter	This study

a American Type Culture Collection.

### Genetic Methods

All oligonucleotides used in this study were given in Table 2. For construction of plasmid pEacsA, the *acsA* gene from *B. subtilis* was amplified from chromosomal DNA by PCR with primer pair acsA1/acsA2. The resulting fragment was digested with SacI and XbaI and ligated into SacI/XbaI-restricted vector pEC-XK99E. For construction of plasmid pEacsAgltA, the *gltA* gene from *C. glutamicum* ATCC 13032 was amplified from chromosomal DNA by PCR with primer pair gltA1/gltA2. The resulting fragment was digested with XbaI and SbfI and ligated into XbaI/SbfI-restricted pEacsA. For construction of plasmid pEacsAgltAsucE, the *sucE* gene from *C. glutamicum* ATCC 13032 was amplified from chromosomal DNA by PCR with primer sucE1/sucE2. The resulting fragment was digested with SbfI and ligated into SbfI-restricted pEacsAgltA. The in-frame deletion of *sdhCAB* operon in *C. glutamicum* ATCC 13032 was achieved via a two-step homologous recombination procedure using the suicide vector pD*sacB.* The flanking regions of *sdhCAB* operon were amplified from chromosomal DNA using primer pairs sdh1/sdh2 and sdh3/sdh4. The flanking fragments were gel purified, mixed in equal amounts, and subjected to crossover PCR. The resulting fusion products containing the upstream and downstream regions were ligated into EcoRI/PstI-restricted pD*sacB* and transformed into *C. glutamicum* ATCC 13032 by electroporation. The procedure of gene deletion was carried out as described previously [17].

**Table 2:** Primers used in this study.

Primers	Sequence
sdh1	ATCGGAATTCTGATGCGCAATAACACCGGTA (EcoRI)
sdh2	*GCGAGTTCTGCGGTTCGCCTCCGTCGTAAT* TTTTCCGTGA
sdh3	*ATTACGACGGAGGCGAACCGCAGAACTCGC* ACTTGACCAC
sdh4	GTAACTGCAGGGCCGGTTTCCTTGACGTAAC (PstI)
acsA1	CTAGGAGCTCAAAGGAGGACAACCATGAACTTGAAAGCGTTACCAG (SacI)
acsA2	CAGCTCTAGATTAATCCTCCATTGTTGACAG (XbaI)
gltA1	AGGGTCTAGACCGTAATCCGGAAGAGTTT (XbaI)
gltA2	ATATCCTGCAGGGTTTCATGCAAAAACGGCCGA (SbfI)
sucE1	CGCGCCTGCAGGACCAAGACCGCTGTTGCAGTG (SbfI)
sucE2	AGATCCTGCAGGTGCGCTTAAGGGGTCAATGC (SbfI)
16srRNA-F1	GGAGAAGAAGCACCGGCTAA
16srRNA-R2	ACGCTCGCACCCTACGTATT
sucE-F1	CTCTCCATCGCCATCTTCAG
sucE-R2	CCGCTTCTTCGGCTTCTT

The underlined nucleotides indicated the restriction site for the appropriate enzymes. The italic nucleotides showed linker sequences for crossover PCR.

### Cultivation Conditions

For the precultivation of *C. glutamicum* ATCC 13032 strains, single clones were grown in 5 mL of modified CGIII medium containing 10 g L^−1^ tryptone, 10 g L^−1^ yeast extract and 42 g L^−1^ 3-morpholinopropanesulfonic acid (MOPS) (pH 7.4) at 30°C and 220 rpm. After overnight incubation, cells of the preculture were inoculated into 500 mL shake flasks containing 50 mL modified CGIII medium supplemented with 55 mM glucose to an initial OD_600_ of 0.06. After inoculation, cultivations for succinate production were performed at 250 rpm and 30°C. For induction, up to 1 mM isopropyl β-D-1-thiogalactopyranoside (IPTG) was added to the culture medium.

The fed-batch cultures were performed in a 1.3-L glass bioreactor (BioFlo 110, New Brunswick Scientific, USA). 50 mL modified CGIII medium supplemented with 55 mM glucose was inoculated with 1 mL frozen stock culture of strain ZX1 (pEacsAgltA) and the culture was incubated on a rotary shaker at 30°C and 250 rpm. Subsequently, the cells were harvested and inoculated into 600 mL modified CGIII medium (pH 7.0) supplemented with about 222 mM glucose to an initial OD_600_ of about 1. The aeration rate was set to 1.0 L min^−1^ and the dissolved oxygen was maintained above 30% saturation level by regulating the stirrer speed from 500 to 1000 rpm. The pH was maintained at 7.0 by automatic addition of the mixture of 6 M potassium hydroxide combined with 2 M potassium carbonate, and the temperature was maintained at 30°C. During the fed-batch process, adequate amounts of 80% (w/v) glucose were injected.

### Analytical Techniques

Extracellular organic acids were measured by HPLC (HP 1100 LC, Agilent Technologies, USA) equipped with a cation-exchange column (HPX-87H, BioRad, USA), an UV absorbance detector (Agilent Technologies, G1315D) and a refractive index (RI) detector (Agilent Technologies, HP1047A). A mobile phase of 2.5 mM H_2_SO_4_ solution at a 0.4 ml min^−1^ flow rate was used and the column operated at 65°C. Succinate and acetate were measured by the RI detector while pyruvate was measured by the UV detector at 210 nm. Glucose concentration was monitored using a SBA sensor machine (Institute of Microbiology, Shandong, China). Growth was determined by measuring the optical density at 600 nm (OD_600_) and one unit of absorbance at 600 nm corresponded to 0.25 g cell dry weight (CDW) per liter. For determination of intracellular acetyl-CoA, 100 mL mid-exponential phase cell culture was taken into precooled centrifuge tubes and centrifuged at 9000×*g* and 4°C for 10 min. The cell pellets were washed twice with 50 mL 100 mM sodium phosphate buffer (pH 3.0, 4°C). Acetyl-CoA was analyzed by HPLC (HP 1100 LC, Agilent Technologies, USA) equipped with a Hypersil ODS (C18) column (125×4 mm, 5 µm, Phenomenex). The sample preparation and the analysis procedure were carried out as previously described [18].

### Enzyme Assays

*C. glutamicum* ATCC 13032 strains were cultivated in modified CGIII complex medium supplemented with 55 mM glucose. At exponential phase, the cells were harvested by centrifugation for 10 min at 9000×*g* and 4°C, washed twice with 15 mL 50 mM Tris-HCl (pH 7.5), centrifuged again and resuspended in the same buffer. The cells were then subjected to sonication for 15 min in an ice bath (130 W, 20 kHz, pulse: 5 s on; 5 s off). Cell debris was removed by centrifugation for 30 min at 10000×*g* and 4°C and the supernatants were used as cell extracts. The acetyl-CoA synthetase activity was determined as previously described [19]. The citrate synthase activity was determined as described elsewhere [20]. Total protein concentrations were determined by the Bradford method using bovine serum albumin as standard. The specific enzyme activities were expressed as units per mg protein.

### Real-time Quantitative PCR (RT-*q*PCR)

*C. glutamicum* ATCC 13032 strains were grown to the exponential phase. Then the cells were harvested by centrifugation for 2 min at 13000×*g* and 4°C and washed once with distilled water. Total RNA was extracted with RNAprep pure Kit DP430 (Tiangen, Beijing, China). Firstly, 500 ng of total RNA was transcribed into cDNA using Quant Reverse Transcriptase with random primers (Tiangen, Beijing, China). Samples were then analyzed using a Light Cycler® 480 II (Roche, Basel, Switzerland) with Real Master Mix (SYBR Green). The 16S rRNA gene was used as reference for normalization and the primers used for gene quantification were list in Table 2.

## Results and Discussion

### Aerobic Succinate Production by Strain ZX1

**Figure 1:**
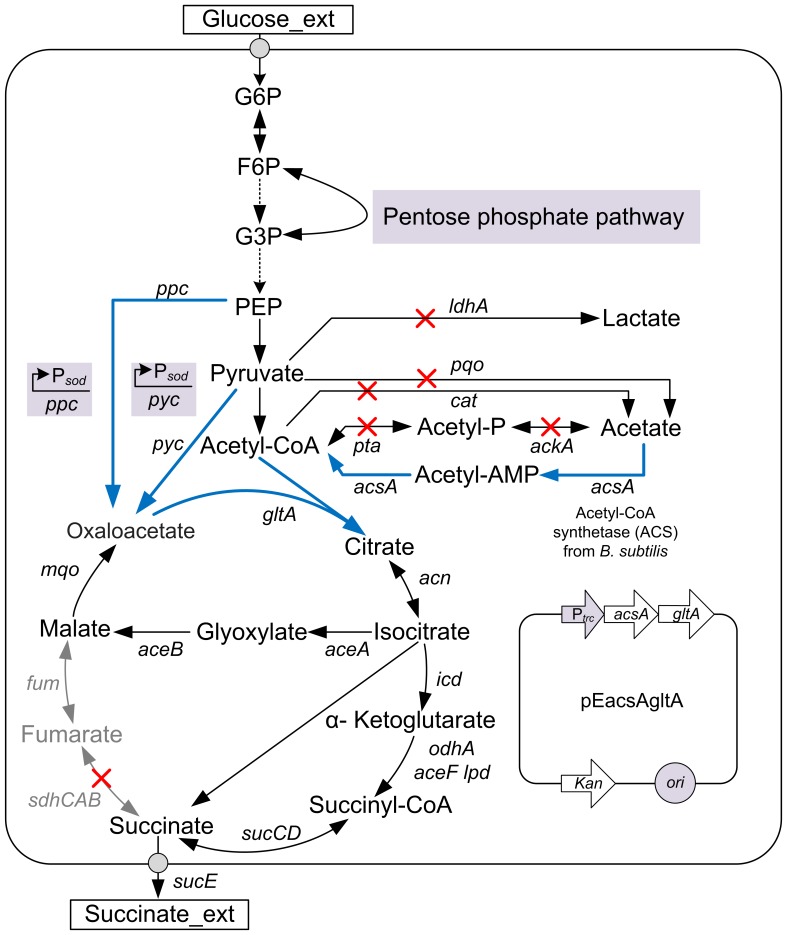
The central metabolic pathways of *C. glutamicum* ATCC 13032 and the engineering strategies for aerobic succinate overproduction. Intermediate pathways omitted in glycolysis were illustrated by broken line. The red X indicated that the pathways were disrupted. The blue arrows indicated that pathways were overexpressed. Relevant reactions were represented by the genes encoding for corresponding enzymes: *ppc*, phosphoenolpyruvate carboxylase; *pyc,* pyruvate carboxylase; *mqo*, malate:quinone oxidoreductase; *fum*, fumarate hydratase; *sdhCAB*, succinate dehydrogenase complex; *aceF lpd odhA*, 2-oxoglutarate dehydrogenase complex; *sucCD*, *succinyl*-*CoA synthetase*, *icd*, isocitrate dehydrogenase; *acn*, aconitate hydratase; *gltA*, citrate synthase; *aceA*, isocitrate lyase; *aceB*, malate synthase; *ldhA*, L-lactate dehydrogenase; *pqo*, pyruvate : quinone oxidoreductase; *pta*, phosphotransacetylase; *ackA*, acetate kinase; *cat*, acetyl-CoA:CoA transferase; *acsA*, acetyl-CoA synthase; *sucE*, succinate exporter. Abbreviations: G6P, glucose-6-phosphate; F6P, fructose-6-phosphate; G3P, glyceraldehyde-3-phosphate; Acetyl-P, acetylphosphate; Acetyl-AMP, acetyladenylate; PEP, phosphoenolpyruvate.

**Figure 2:**
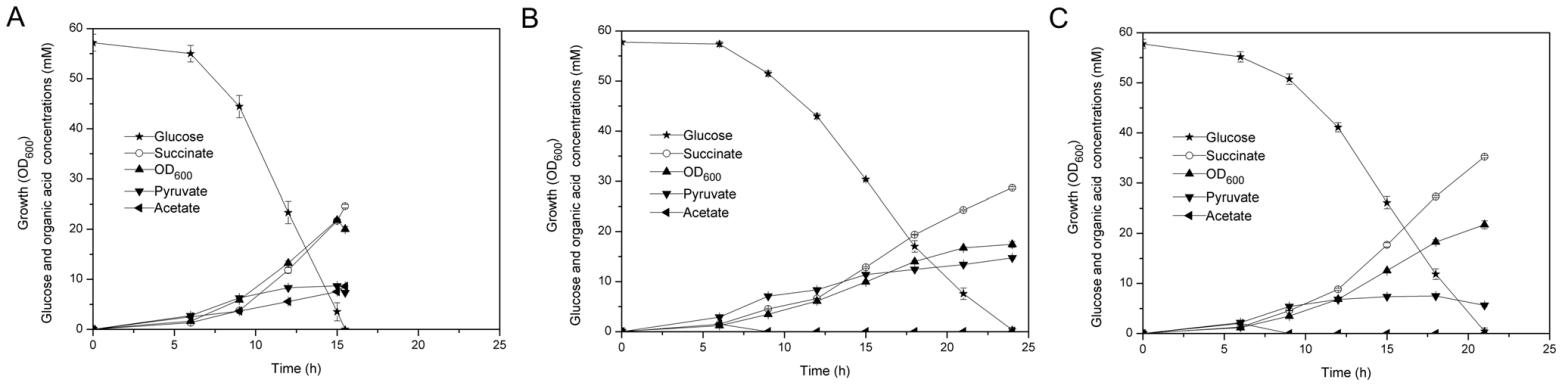
Physiological features of succinate-producing *C. glutamicum* ATCC 13032 strains in batch cultures. (A) ZX1, (B) ZX1 (pEacsA) and (C) ZX1 (pEacsAgltA). The data represented average values of three independent cultures with corresponding standard deviations.

Efforts to increase succinate production by *C. glutamicum* ATCC 13032 under aerobic conditions have been made by disrupting the succinate dehydrogenase complex and pathways leading to the synthesis of acetate, as well as co-overexpressing pyruvate carboxylase and PEP carboxylase [13]. Similarly, starting with the wild-type ATCC13032, the genes of *pqo*, *pta* and *cat* were disrupted, and both of the native promoters of genes of *ppc* and *pyc* were replaced by the strong promoter of the superoxide dismutase (*sod*) (Figure 1). Also, the *ldhA* gene encoding L-lactate dehydrogenase was inactivated to avoid lactate accumulation in further strain improvement. Finally, the deletion of *sdhCAB* operon was introduced, yielding strain ZX1. To test its ability to produce succinate from glucose, shake-flask cultivations were carried out in modified CGIII medium with 55 mM glucose at 30°C and 250 rpm. In order to prevent acidification, 3-morpholinopropanesulfonic acid (MOPS) was added as a buffering agent. As described in Figure 2A and Table 3, strain ZX1 consumed glucose completely within 16 h and showed a growth rate of 0.35 h^−1^. Also, it produced 24.60 mM succinate at the end of the cultivations with a succinate yield of 0.43 mol (mol glucose) ^−1^, which exhibited a 3.07-fold increase compared to the *sdhCAB*-dificient mutant. The specific succinate productivity was 0.94 mmol g_CDW_^−1^ h^−1^. In addition, 7.33 mM pyruvate and 8.59 mM acetate were accumulated in the supernatant.

**Table 3:** Relevant process parameters of aerobic batch cultures of various *C. glutamicum* ATCC 13032 strains.

Strain	Growth rate(h^−1^)	Glucose consumption (mM)	Specific succinate productivity (mmol g_CDW_^−1^ h^−1^)	Succinate yield (mol(mol glucose) ^−1^)	Succinate (mM)	Acetate (mM)	Pyruvate (mM)
Δ*sdhCAB*	0.33±0.01	56.11±0.79	0.35±0.01	0.14±0.00	7.88±0.10	48.34±0.72	ND
ZX1	0.35±0.00	57.22±1.67	0.94±0.12	0.43±0.01	24.60±0.35	8.59±0.16	7.33±0.00
ZX1(pEC-XK99E)	0.32±0.02	58.28±1.72	1.03±0.01	0.43±0.02	25.06±0.26	8.99±0.20	7.67±0.09
ZX1(pEacsA)	0.27±0.00	57.41±0.52	1.20±0.01	0.50±0.01	28.67±0.24	ND	14.75±0.19
ZX1(pEacsAgltA)	0.30±0.00	57.31±0.58	1.26±0.02	0.61±0.00	35.24±0.25	ND	5.64±0.69
ZX1(pEacsAgltAsucE)	0.29±0.01	56.40±1.49	1.25±0.02	0.62±0.02	34.97±0.57	ND	5.69±0.23

The growth rate and specific succinate productivity were calculated within the exponential phase of the cultivations. The product concentration was determined at the end of cultivations when glucose was consumed. Values were given as the averages and standard deviations of three independent cultures. ND, not detected.

### Introduction of a Heterologous Acetate Assimilation Pathway

Despite that all known genes responsible for acetate production were inactivated, acetate still accumulated. The remaining acetate formation pathways were still unclear. Several genes of *C. glutamicum* ATCC 13032 were annotated as putative acetyltransferases, hydrolases or dehydrogenases and could be involved in the conversion of acetyl-CoA to acetate [13], [21], [22]. Inactivation of one or more of these genes might reduce acetate formation, whereas the influence was uncertain [13], [21]. Alternatively, introduction of an acetate assimilation pathway could address this conundrum. Even though *C. glutamicum* ATCC 13032 utilized the native acetate kinase-phosphotransacetylase (PTA-ACK) pathway to uptake acetate under aerobic conditions [18], this reversible pathway contributed to acetate formation in the *sdhCAB*-deficient mutant [13], [21], which produced significant amounts of acetate (Table 3). Unlike *C. glutamicum*, *B. subtilis* utilized another acetate assimilation pathway catalyzed by AMP-forming acetyl-CoA synthetase (ACS) via two enzymatic steps (Figure 1), and the PTA-ACK pathway was not required for acetate utilization but was required for its formation when grown in the presence of an excess of glucose [23]. Moreover, ACS was reported to have higher affinity for substrate acetate than PTA-ACK [23].

Accordingly, the ACS from *B. subtilis* was selected and expressed in strain ZX1, resulting in strain ZX1 (pEacsA). Because the expression of acetyl-CoA synthetase (ACS) in *C. glutamicum* ATCC 13032 has never been accomplished, crude extract enzyme assay was performed. The specific ACS activity of 8.03±0.12 mU mg-protein^−1^ was verified in strain ZX1 (pEacsA) whereas no activity was detected with the empty plasmid (pEC-XK99E), indicating its functional expression in this host. In batch cultivations (Figure 2B and Table 3), strain ZX1 (pEacsA) showed lower growth rate (0.27 h^−1^) compared to strain ZX1. In addition, there was no accumulation of acetate at the end of cultivations. In the case of succinate production, this strain produced 28.67 mM succinate at the end of cultivations and showed a slight increase in succinate yield (0.50 versus 0.43 mol (mol glucose) ^−1^). The specific succinate productivity of strain ZX1 (pEacsA) was 1.20 mmol g_CDW_^−1^ h^−1^, which was 28% higher than that of strain ZX1. Also, 14.75 mM pyruvate was formed with a yield of 0.26 mol (mol glucose) ^−1^. These results demonstrated that exogenous ACS expression in succinate-producing *C. glutamicum* ATCC 13032 was an efficient approach to cope with acetate accumulation.

### Reduction of Pyruvate Accumulation by Overexpressing Citrate Synthase

**Figure 3:**
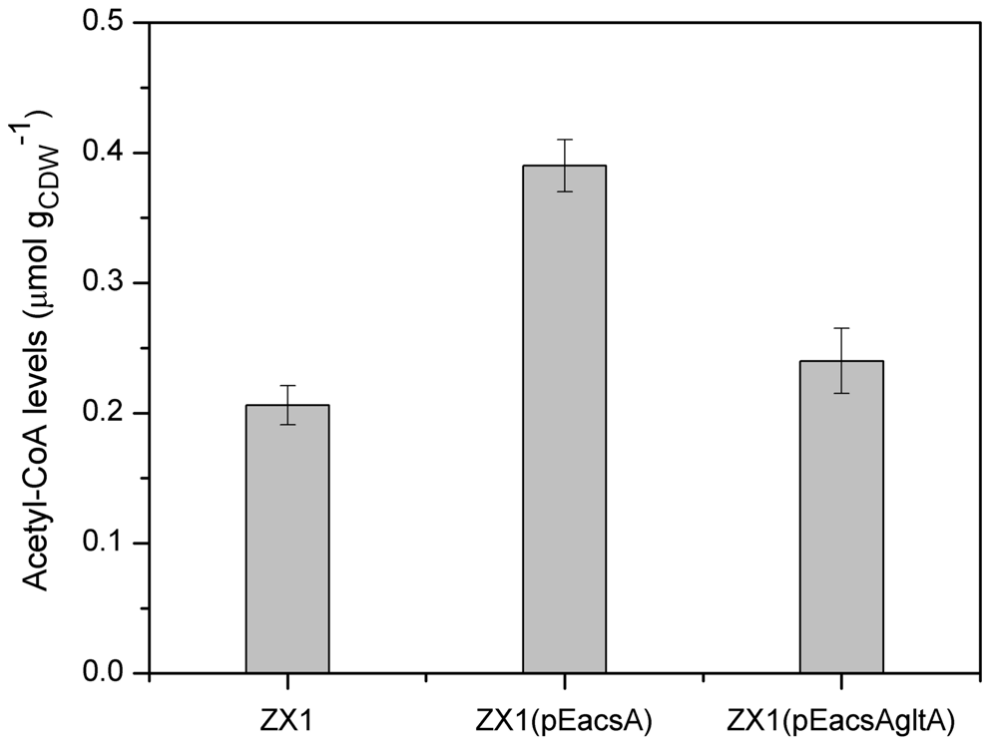
Changes in the levels of intracellular acetyl-CoA. The data represented average values of two independent cultures with corresponding standard deviations.

As described above, strain ZX1 (pEacsA) showed restricted growth and accumulated pyruvate as the major byproduct. In *E*. *coli*, inactivating acetate formation pathways led to reduced cell growth and/or increased pyruvate accumulation, which were attributed to the accumulation of intracellular metabolic intermediates, such as acetyl-CoA [24], [25]. Thus, it could be that intracellular acetyl-CoA of strain ZX1 (pEacsA) would accumulate, especially in the presence of an acetate assimilation pathway as well as the absence of major acetate formation pathways (Figure 1). It was also reported that activity of the pyruvate carboxylase, which contributed over 90% of the oxaloacetate replenishment when grown on glucose, was inhibited by acetyl-CoA (110 µM) [26]. Actually, intracellular metabolites analysis revealed that the level of acetyl-CoA of strain ZX1 (pEacsA) was 89% higher than that of strain ZX1 (Figure 3), which could explain the phenomena of higher accumulation of extracellular pyruvate and reduced growth rate with strain ZX1 (pEacsA) (Figure 2B and Table 3). Recently, Lin et al [27] developed an efficient aerobic succinate-producing *E. coli* HL27659K and also found that this strain had high-level citrate synthase activity. In contrast, a lysine-overproducing mutant of *C. glutamicum* was reported to have low-level citrate synthase activity, which represented low flux towards TCA cycle [28].

Thus, in order to alleviate the accumulation of intracellular acetyl-CoA and pull more carbon towards succinate synthesis, the native citrate synthase encoded by *gltA* gene was overexpressed by introducing plasmid pEacsAgltA into strain ZX1, resulting in strain ZX1 (pEacsAgltA). The specific CS activity of this strain was 4.51±0.09 U mg-protein^−1^, which exhibited about six-fold increased activity compared to strain ZX1 (pEacsA) (0.69±0.03 U mg-protein^−1^), indicating its functional overexpression. In subsequent cultivations, the cell growth rate and organic acids production, as well as intracellular acetyl-CoA level were analyzed. As described in Figure 2C and Table 3, this strain showed a growth rate of 0.30 h^−1^ and accumulated 5.64 mM pyruvate with a yield of 0.10 mol (mol glucose) ^−1^, which was 62% lower compared to strain ZX1 (pEacsA). Also, the succinate yield of 0.61 mol (mol glucose)^−1^ and the specific succinate productivity of 1.26 mmol g_CDW_^−1^ h^−1^ were increased by 22% and 5% compared to strain ZX1 (pEacsA), respectively. In addition, the intracellular level of acetyl-CoA was decreased by 38% after introducing this modification (Figure 3). These results indicated that enhanced expression of the citrate synthase was effective for the efficient production of succinate under aerobic conditions.

#### Effect of modifying export system on aerobic succinate production

**Figure 4:**
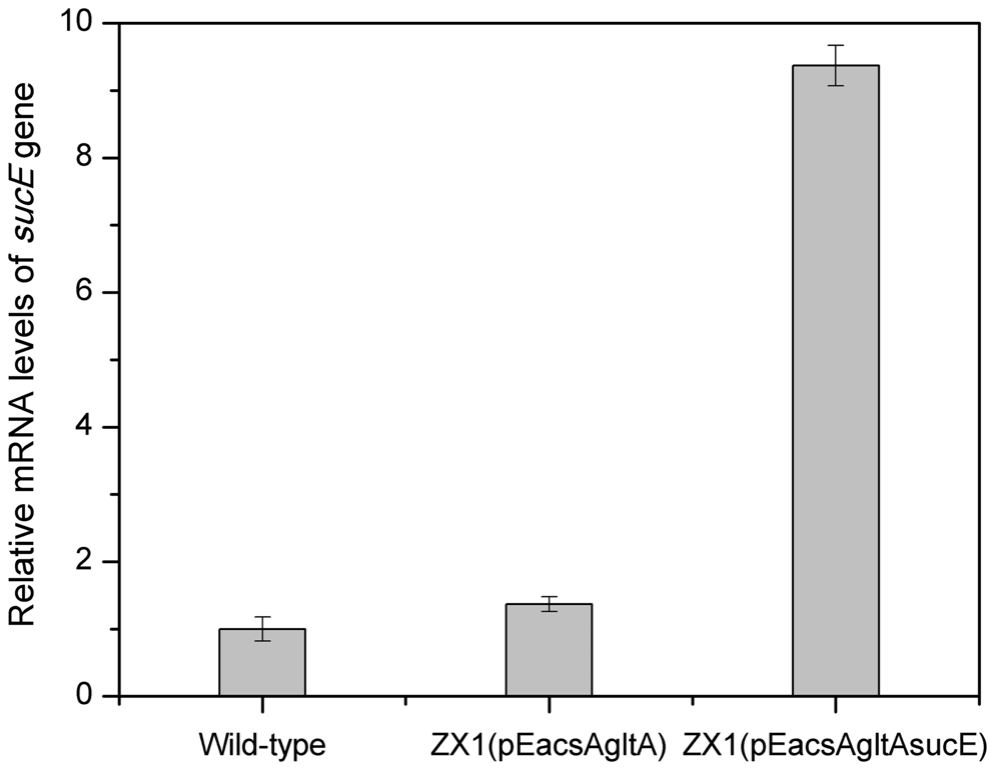
Changes in the transcript levels of *sucE* gene. The average value of wild-type *C. glutamicum* ATCC 13032 was set as 100%. The data represented average values of two independent cultures with corresponding standard deviations.

In *C. glutamicum* ATCC 13032, several C4-dicarboxylic acid transporters have been identified. The *dccT* and *dctA* genes were responsible for C4-dicarboxylate uptake under aerobic conditions [29], [30], while the *sucE* gene was responsible for succinate export under anaerobic conditions [31]. To examine whether succinate production could be further increased by *sucE* overexpression, plasmid pEacsAgltAsucE was introduced into strain ZX1, resulting in strain ZX1 (pEacsAgltAsucE). As expected, the *sucE* expression level of this strain was significantly increased by nearly seven-fold compared to strain ZX1 (pEacsAgltA), reflecting its successful overexpression (Figure 4). In batch cultivations, strain ZX1 (pEacsAgltAsucE) showed a growth rate of 0.29 h^−1^, whereas the succinate yield and specific succinate productivity were almost unchanged compared to strain ZX1 (pEacsAgltA) (Table 3), indicating that the expression level of this gene or the ability of other export systems [31] might be sufficient under this condition.

#### Fed-batch cultures for aerobic succinate production

**Figure 5:**
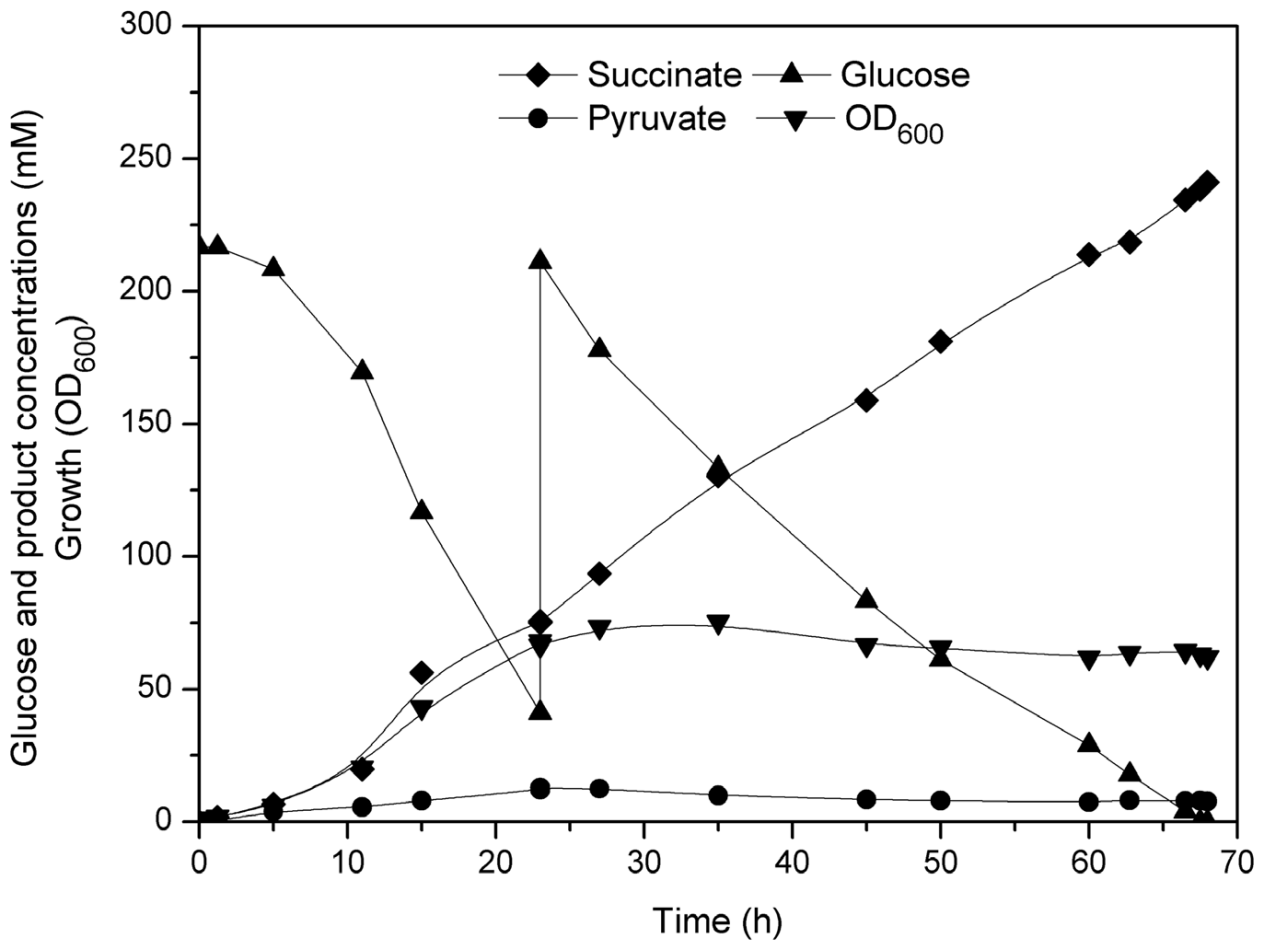
Fed-batch cultures of strain ZX1 (pEacsAgltA) for succinate production. Two independent fed-batch cultures were performed, both revealing comparable results.

In order to evaluate the suitability of strain ZX1 (pEacsAgltA) as a host for the aerobic succinate production with higher level, fed-batch cultures were carried out in modified CGIII medium, initially containing 222 mM glucose. The detailed results of the fed-batch cultivation were described in Figure 5. After the glucose concentration was below 55 mM, adequate amounts of 80% glucose (w/v) were added at 23 h. Strain ZX1 (pEacsAgltA) grew to a maximum OD_600_ of 75 within 35 h. The succinate concentration continuously increased to 76 mM within 23 h, resulting in a volumetric productivity of 3.29 mM h^−1^. The volumetric productivity during the second stage (after glucose feeding) was further increased to 3.69 mM h^−1^. The overall succinate yield was 0.63 mol (mol glucose) ^−1^. The pyruvate yield obtained in bioreactor (0.02 mol (mol glucose) ^−1^) was lower compared to that obtained in shake flask (0.10 mol (mol glucose) ^−1^). It could be due to the different dissolved oxygen levels between bioreactor and shake flask. In shake flask, the cells faced oxygen limitation when OD_600_ was above 10 [32], while the oxygen was surplus (above 30% saturation level) in bioreactor. Recently, Wieschalka et al [17] described a similar observation for a pyruvate production process with *C. glutamicum* ATCC 13032. They found that surplus oxygen could reduce pyruvate production and the physiological state of the cells had a significant impact on the overall production behavior. Taken together, these results indicated that strain ZX1 (pEacsAgltA) was a useful platform for the production of succinate under aerobic conditions.

### Conclusions

In summary, this work demonstrated that introduction of an acetate assimilation pathway from *B. subtilis* was an efficient approach to reduce acetate accumulation in succinate-producing *C. glutamicum* ATCC 13032. It was also shown that high level expression of citrate synthase could assure efficient production of succinate under aerobic conditions. To the best of our knowledge, the succinate titer, yield and productivity obtained in fed-batch cultivations with strain *C. glutamicum* ATCC 13032 ZX1 (pEacsAgltA) are the highest currently reported with *Corynebacterium* species under fully aerobic conditions. Moreover, The complete elimination of byproduct acetate in the production process and the high succinate productivity with strain *C. glutamicum* ATCC 13032 ZX1 (pEacsAgltA) offer advantages over other microorganisms, making it a promising platform for the aerobic production of succinate [3], [12], [14], [15], [16]. Even so, further strain improvement and optimization of culture conditions will be pursued to make better performance.
